# Music we move to: Spotify audio features and reasons for listening

**DOI:** 10.1371/journal.pone.0275228

**Published:** 2022-09-29

**Authors:** Deniz Duman, Pedro Neto, Anastasios Mavrolampados, Petri Toiviainen, Geoff Luck

**Affiliations:** Department of Music, Art and Culture Studies, Centre of Excellence in Music, Mind, Body and Brain, University of Jyväskylä, Jyväskylä, Finland; University of Western Ontario, CANADA

## Abstract

Previous literature has shown that music preferences (and thus preferred musical features) differ depending on the listening context and reasons for listening (RL). Yet, to our knowledge no research has investigated how features of music that people dance or move to relate to particular RL. Consequently, in two online surveys, participants (N = 173) were asked to name songs they move to (“dance music”). Additionally, participants (N = 105) from Survey 1 provided RL for their selected songs. To investigate relationships between the two, we first extracted audio features from dance music using the Spotify API and compared those features with a baseline dataset that is considered to represent music in general. Analyses revealed that, compared to the baseline, the dance music dataset had significantly higher levels of energy, danceability, valence, and loudness, and lower speechiness, instrumentalness and acousticness. Second, to identify potential subgroups of dance music, a cluster analysis was performed on its Spotify audio features. Results of this cluster analysis suggested five subgroups of dance music with varying combinations of Spotify audio features: “fast-lyrical”, “sad-instrumental”, “soft-acoustic”, “sad-energy”, and “happy-energy”. Third, a factor analysis revealed three main RL categories: “achieving self-awareness”, “regulation of arousal and mood”, and “expression of social relatedness”. Finally, we identified variations in people’s RL ratings for each subgroup of dance music. This suggests that certain characteristics of dance music are more suitable for listeners’ particular RL, which shape their music preferences. Importantly, the highest-rated RL items for dance music belonged to the “regulation of mood and arousal” category. This might be interpreted as the main function of dance music. We hope that future research will elaborate on connections between musical qualities of dance music and particular music listening functions.

## Introduction

Throughout the lifespan, people interact with music for different reasons across a variety of listening situations [1]. Music can be used for entertainment, relaxation, improving mood, self-awareness, forming and maintaining relationships, feeling connected, diversion, and dancing, as well as to accompany daily human activities such as exercising, partying, commuting, sleeping, and so on [2, 3]. Moreover, musical preferences and associated reasons for listening (RL) have been reported to differ depending on factors such as personality traits [4], listening habits [5], and age [3]. In a study of motivations for listening to music and contexts in which that listening took place, Boer & Fischer [6] proposed that listening to music serves six main functions: background listening, memories, diversion, emotional regulation, self-reflection and social bonding. A more recent study narrowed these down to three main functions: regulation of arousal and mood, achievement of self-awareness, and expression of social relatedness [2]. All of these functions can be interpreted as what music broadly offers listeners and in what possible ways people listen to music.

### Music listening as an immersive, affective, movement-inducing and social experience

It is notable that, in some languages such as Sanskrit, Thai and Igbo of Nigeria, there is no clear terminological distinction between music and dance, being understood as aspects of the same “musical activity” [7]. Ancient musical activities were part of daily rituals (such as funeral ceremonies, hunting, or rain dancing) and are shown to have the functions of establishing and expressing social identity, evoking and regulating emotions, promoting social bonding, group cohesion, and prosocial behaviour. Music and dance are even argued to have originated together [8]. While pinpointing the birth of music and dance is challenging, it can certainly be said that music and dance share some common functions in daily life.

Music is a powerful driver of affect- and motion-related experiences. Music listening frequently creates feelings of pleasure, and induces spontaneous movements such as foot-tapping, swaying or head nodding [9]. The fact that even infants move to music rather automatically [10] demonstrates the interwovenness of dance and music. Moreover, in the absence of overt movements (e.g., during passive listening), particularly in rhythm and beat perception, it is not only auditory regions of the brain that are activated; motor [11–13], and reward [14] regions also fire. Thus, one might describe the perception of music as a pleasurable and movement-inducing state.

The popular musical term, *groove*, commonly understood as a pleasurable desire to move to music [15] has recently been more comprehensively described as a *multifaceted participatory experience* [16]. More specifically, Duman and colleagues [16] argue that an experience of groove is not only connected with movement and positive affect but also associated with sensations of immersion and social connectedness shaped by a delicate interaction of specific music- performance-, and individual difference-related variables. Previous groove literature reported several intra- and extra-musical variables relate to groove experience. One recent study has reported that there are “different types of groove” experiences depending on musicians’ strategies [17] which give rise to certain musical features [18, 19] such as clear pulse and meter [20], low frequency range [19], tempo around 100–120 beats per minute (bpm) [21], medium levels of syncopation [9], and harmonic complexity [22] being associated with higher groove ratings (mainly based on the induced experiences of pleasure and desire to move). Experiences of flow [23] and social connection [24] have received some attention, too. Other work has demonstrated the role individual differences such as musical preferences and familiarity influence the experience of groove [25]. Additionally, although pop and funk musical styles in particular have been linked with high groove ratings, groove is also associated with other genres, including classical music [26].

While there is a growing amount of research investigating the concept of groove, to the best of the present authors’ knowledge, no research has examined the influence of listener’s current state, goals, or RL on the experience of groove. Thus, questions such as why and how people move to music remain open to further exploration. Our focus in this paper was to better understand why people choose to listen to specific music for dancing as a function of both musical features and RL. Before describing the current study in detail, we summarise literature that has examined connections between musical features and RL.

### Literature on Spotify audio features and reasons for listening

One focus of such research has been on retrieving musical information from songs on playlists designed with certain moods or listening situations in mind. Since we employed Spotify audio feature analysis in the current study, we focus here on previous research that has utilised a similar method. This approach has revealed that such playlists do indeed vary in terms of their Spotify audio features [27]. Using Spotify data, recent research demonstrated that people’s musical preferences follow a certain pattern depending on the period of the day [28, 29]. In other words, Spotify audio features of music that people listen to fluctuate throughout the diurnal cycle. Musical preferences are characterized in the morning by high loudness, valence and energy, in the afternoon by an increase in tempo, beat strength and danceability, and during the night by the lowest values of loudness and tempo [28]. What’s more, a moderately significant correlation between diversity of human activity during the day and variability in the average Spotify audio features has been identified [28]. This suggests that Spotify audio features of music people choose to listen to vary depending on the activity they engage with. For instance, one study investigated songs that are used for sleeping [30]. Such songs were found to be generally softer, slower and often instrumental compared to general music. However, results of a cluster analysis revealed six different subgroups of sleep music, including some with songs that are fast, loud, and energetic. This surprising finding was interpreted as a possible explanation for various motivations for listening to music. Other work has linked musical preferences with stress management and emotional coping strategies during the COVID-19 pandemic [31, 32] and with patients’ pain management [33]. In particular, Spotify audio features such as high valence, energy, danceability, tempo and low instrumentalness were found to correlate with the music people choose associated with emotional coping mechanisms. These studies exemplify how certain Spotify audio features and reasons or functions of listening to music are associated.

### The current study

Despite the growing amount of research that links musical features with certain listening contexts and reasons, no study appears to have investigated connections between qualities or features of music that people choose for dancing and RL. Thus, our aim was to investigate Spotify audio features of dance music, and whether these features are homogenous or reveal different types of such music as determined by different combinations of audio features. Note that “dance music” in this paper refers to music which people dance / move to, not any specific commonly-understood genre or style.

We hypothesised that a dance music dataset will exhibit higher levels of Spotify audio features such as danceability, energy, and valence compared to a baseline dataset. Additionally, in a similar vein to the work on sleep music by Scarratt et al. [30], we expected to identify several subgroups of dance music, especially in light of the different ways and styles in which people move to music. Moreover, we explored other reasons why people listen to music for dancing, in particular with the aim of differentiating dance-related listening reasons from more general reasons. Considering the various factors influencing people’s musical preferences associated with RL, we expected to observe a variation in the music people dance to associated with their particular listening purpose. Finally, we explored whether Spotify audio features of dance music can be linked with specific RL. We hypothesised that certain subgroups of dance music would be more suitable for specific RL. We thus sought answers to four principal research questions:

1. What are the general Spotify audio features of dance music?
2. Does all dance music have similar characteristics, or can we identify multiple subgroups of dance music, each with different combinations of Spotify audio features?
3. Can the music people choose to dance to be associated with other typical RL?
4. Can Spotify audio features of dance music be related to people’s RL?

To answer these questions, we conducted two online surveys in which we asked participants to name songs that they “want to move to” and rate these songs in terms of their associated RL.

## Method

### Participants

#### Survey 1

One hundred and five participants (61 women, 41 men, 3 other) aged 16 to 54 (*M* = 27.07, *SD* = 6.46) completed the entire survey. Participants originated from 19 different countries, with the majority of them reporting to be Finnish (*N* = 56) or Turkish (*N* = 23) nationals.

#### Survey 2

Sixty-eight participants (39 women, 28 men, 1 other) aged 14 to 53 (*M* = 29.34, *SD* = 7.88) completed the survey. Participants originated from 21 different countries, with the majority of them reporting to be from the USA (*N* = 14), Turkey (*N* = 9) and Finland (*N* = 9).

### Procedure and materials

#### Survey 1

Data were gathered as part of the online listening survey described in Duman et al. [16]. For the present study, participants rated their *general* music listening habits using a questionnaire containing 21 RL items. These items were gathered and adapted mainly from two papers: (1) by Schäfer and colleagues [2] which provides a comprehensive review of functions of music listening literature, and (2) by Randall & Rickard [34] which presents brief use of RL items in research utilising the experience sampling method. Responses were given on a 5-point likert scale (never—rarely—sometimes–often–very often). In addition, they were asked to “give an example song which ‘makes you want to move’” and subsequently “select all the reasons why you listen to the song you provided above in your daily life” from the 21 RL items (as boolean: true/false). Hereafter, these ratings will be denoted as *RL general* and *RL dance*, respectively.

#### Survey 2

In a separate online survey, in order to increase the sample size for musical analysis of the provided songs, participants were asked to “give 3 different song examples which ‘make you want to move’”. Additionally, demographic information was collected, but no RL data were gathered. All participants declared that they had not taken part in *Survey 1*.

In both surveys, data were collected on webropol.com via personal social media accounts and University of Jyväskylä emailing lists. Participants were informed about the content of the survey, their rights as a participant, and were requested to provide their written consent to participate. Participation and data processing were anonymised. According to the University of Jyväskylä’s guidelines, no further ethical approval was required as the study did not have a potential risk for participants.

## Analysis

### General characteristics of dance music

In order to determine common Spotify audio features of dance music, data representing music in general were gathered from 3.706.623 songs in the Music Streaming Sessions Dataset (MSSD) [35] to use as a baseline against which to compare the dance music dataset consisting of 278 unique songs named by participants. The dance music dataset is available under the Spotify playlist ID: 7hLTtnu2eJXuYTYhVxPe2m. Since several songs were named more than once, the repeating songs (*N* = 13) were included only once in this dataset. Additionally, as the Spotify API was used for analysis, songs that were not found on Spotify (*N* = 12) were also excluded. While the Spotify audio features can be found from MSSD dataset, to extract dance music dataset’s Spotify audio features, we modified a Python script called “GeneralizedSpotifyAnalyser” provided by Ole Adrian Heggli [30]. Spotify audio features pertaining to energy, acousticness, danceability, valence, loudness, instrumentalness, speechiness, liveness and tempo were then gathered for both the baseline and dance music datasets. Note that, since Spotify does not provide extensive descriptions of how these features are calculated (as also discussed by Heggli and colleagues [30]), the exact meaning and calculation method of these features are debatable. Spotify does provide brief description of these features which can be found in S1 Text. Furthermore, while Spotify uses “audio features” as a general term, they are not purely audio properties of music. For instance, while loudness can be seen as an audio feature, danceability relates to the use of music. Spotify states the following categorization for these features: “mood” for danceability, valence, energy, and tempo; “properties” for loudness, speechiness, and instrumentalness; and “context” for liveness and acousticness as described in Features | Spotify for Developers [36]. To be consistent with their general terminology, in this paper we refer to those extracted features as “Spotify audio features”.

A series of two sample t-tests were applied to each Spotify audio feature comparing baseline and dance music datasets. Due to unequal variance between samples, *Welch’s* t-test method was adopted. The t-tests were later corrected for multiple comparison error with a conservative -*Bonferroni-* method (α = .01). Additionally, effect sizes were calculated using *Cohen’s d*. *Cohen’s d* effects sizes are commonly interpreted in behavioural sciences as small (d = 0.2), medium (d = 0.5) and large (d = 0.8) [37]. Given its size, the baseline dataset was treated as a population of the dance music dataset and we applied one-way t-tests for comparison. The results of these one-way t-tests revealed similar findings compared with two sample’s Welch’s t-tests, thus we opted for only reporting the results of two sample t-tests.

### Subgroups of dance music

In order to investigate homogeneity of dance music characteristics, we ran a *k-means clustering analysis* on the audio features of the 278 dance music songs. An *elbow analysis* was used to determine the number of clusters and showed optimal results for solutions with 4–6 clusters. For the clustering analysis, data were standardized and the maximum iteration parameter was set to 3000. Three separate k-means clustering analyses were run, one for each of the candidate optimum number of clusters (k = 4–6). The results were compared in terms of their distributions (the percentage of the data that each cluster had) and the interpretability of each cluster. The 5-cluster solution was deemed optimal and subsequently selected.

### Reasons for listening

#### Comparing RL general and dance ratings

To investigate people’s RL associated with dancing, our third analysis focused on participants’ *RL general* and *dance* ratings. All subsequent analyses were based on the data collected in Survey 1 concerning 100 dance songs as the RL data were collected only in Survey 1. Taking the scale difference between the two ratings into account, the data were first z-scored and then a comparison between *RL general* and *dance* was made by plotting the frequency distribution of the z-scored ratings. Additionally, to reveal differences between *RL general* and *dance* ratings further, ratings of RL items were rank ordered (from 1 to 21; 1 representing the highest, 21 the lowest ranks) and differences in ranks were calculated by subtracting *RL general* and *dance* ranks for each item.

#### Factor analyses

Next, in order to deepen our comparison of *RL general* and *dance*, we employed factor analyses to reduce dimensionality of the data using Python’s “factor_analyzer” package with *method* set to ‘principal’ and *rotation* set to ‘promax*’*. This was the preference as we expected the latent variables (factors) to be correlated with each other. The most meaningful number of principal components was determined visually through *Cattell’s scree test* (1966) [38], and statistically through *Velicer’s Minimum Average Partial test* (MAP) [39]. These tests are reported to be among the most reliable graphic and numeric based methods, respectively [39, 40].

### Relating RL to subgroups of dance music

For the purpose of relating subgroups of dance music (determined by clusters of Spotify audio features) to participants’ *RL dance* ratings, we plotted percentages of *RL dance* item ratings for each subgroup of dance music as a heatmap. The percentage calculation was made by dividing the actual *number of total selections* with *possible (maximum) number of total selections* (which is equal to all songs, *N* = 100).

## Results

### General characteristics of dance music

**Fig 1:**
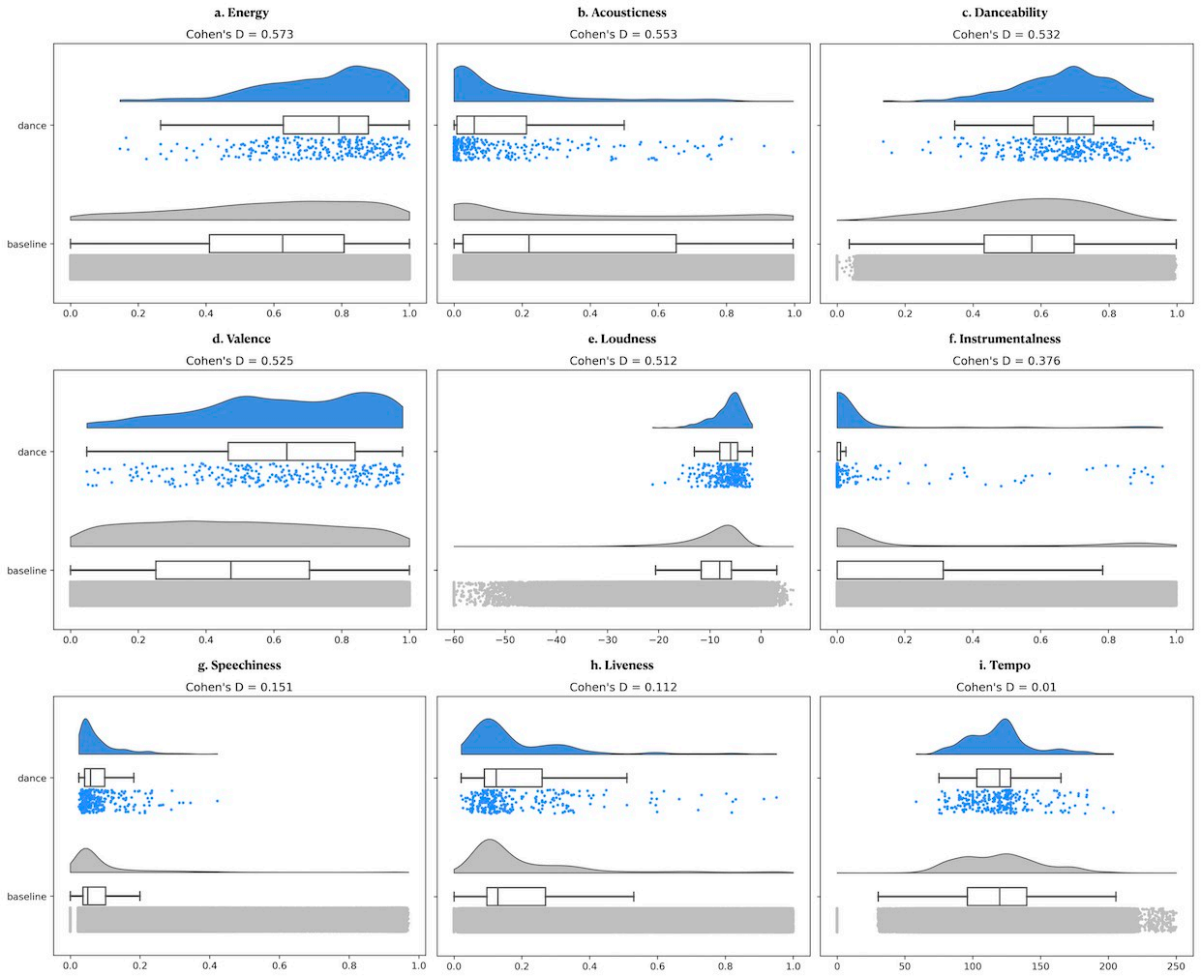
Spotify audio features comparing baseline (grey) and dance (blue) music datasets. Two sample’s t-tests revealed significant differences for all features except for liveness (h) and tempo (i). Calculated with Cohen’s *d*, medium effect sizes for energy (a), acousticness (b), danceability (c), valence (d), loudness (e), instrumentalness (f); small effect size for speechiness (g) were found. An additional K-S test revealed significant difference between dance and general datasets’ tempo feature (i). To summarise, dance music can be differentiated from the music in general to be typically around 120 bpm, with higher energy, danceability, valence, loudness, and lower acousticness, instrumentalness, and speechiness Spotify audio features.

The t-tests on Spotify audio features of the *dance* and *general* music datasets revealed significant differences for all features except liveness and tempo. Medium effects (as measured with *Cohen’s d*) were observed for energy, acousticness, danceability, valence, and loudness, and a low effect for speechiness (See Fig 1a–1i). Although the result of the t-tests yielded no statistical difference between the dance and baseline dataset in terms of liveness and tempo (Fig 1h and 1i), with visual inspection, one can suspect that there might be significant differences between the two datasets for tempo (Fig 1i). Therefore, *Kolmogorov—Smirnov (K-S) goodness of fit* tests were applied for each feature. K-S is a non-parametric test that focuses on the maximum difference between two distributions [41]. While result of the K-S test indicated no difference in distributions for liveness, all other features, including tempo, revealed significantly different distributions between dance and baseline datasets. All of the statistics can be found in Table 1. As hypothesized, compared with the baseline, the dance music dataset has higher levels of energy, danceability, valence, and loudness. Moreover, the dance music dataset is characterized by lower levels of acousticness, instrumentalness and speechiness. Importantly, despite both dance music and baseline datasets having average tempi around 120 bpm (Fig 1i), deviation from the average tends to be smaller for the dance music dataset, suggesting that music people dance to is more tightly clustered around 120 BPM.

**Table 1:** Results of the two samples’ Welch’s t-tests, Cohen’s *d* and Kolmogorov-Smirnov tests between the dance and baseline music datasets.

Spotify Audio Features	Dance Music Dataset (N = 278)		Baseline Dataset (N = 3.706.623)		Welch’s t-test		Cohen’s *d*	Kolmogorov-Smirnov test	
	Mean	Standard Deviation	Mean	Standard Deviation	*t—*value	*p—*value	*d*—value	Statistics	*p*—value
Danceability	.66	.14	.56	.19	*t*(277) = 11.61	< .001	0.53	.26	< .001
Energy	.74	.18	.59	.26	*t*(277) = 13.92	< .001	0.57	.26	< .001
Loudness	-6.7	3.07	-9.6	5.73	*t*(277) = 15.95	< .001	0.51	.28	< .001
Speechiness	.08	.07	.11	.14	*t*(277) = -5.37	< .001	0.15	.11	< .01
Acousticness	.16	.21	.35	.34	*t*(277) = -14.83	< .001	0.55	.27	< .001
Instrumentalness	.08	.21	.21	.35	*t*(277) = -10.24	< .001	0.38	.28	< .001
Liveness	.19	.17	.21	.19	*t*(277) = -2.18	= .30	0.11	.07	= .14
Valence	.62	.24	.48	.27	*t*(277) = 9.87	< .001	0.53	.25	< .001
Tempo	119.78	24.25	120.07	30.43	*t*(277) = -0.20	= 1	0.01	.14	< .001

Please note that *p*—values are corrected using the *Bonferroni* method.

To provide a general picture of the kinds of songs participants reported a desire to move to, we present 30 songs from the dance music dataset with the highest Spotify danceability scores in descending order in Table 2. The song *Macarena* by Los Del Rio has the highest Spotify danceability score in our dance music dataset. This song is known by many people for its song-specific dance routine, and is tagged as Tropical by Spotify. In contrast, the song with the second-highest danceability score, *Thunderstorm* by Boris Brejcha, is less well known, and is tagged as German house / techno by Spotify. The list also includes several classic tracks produced in the 1970s and 1980s associated with disco, R&B, funk, and motown (e.g., *Funkytown* by Lipps Inc., *People Get Up and Drive Your Funky Soul* by James Brown, and *Get Down On It* by Kool & The Gang) as well as newer tracks produced after the turn of the millennium associated with genres like dance pop, electronic, and R&B (e.g., *Treasure* by Bruno Mars, *Lose Yourself to Dance* by Daft Punk & Pharrell Williams, and *Shape of You* by Ed Sheeren). Moreover, the list is not limited to popular songs and typical genres; it also includes salsa, Latin and Brazilian jazz styles, as well as culture-specific songs. All in all, this variety led to the next analysis for which we sought possible types of dance music.

**Table 2:** 30 songs from the dance music dataset with the highest Spotify danceability scores.

	Song Name	Artist Name	Genre
1	Macarena	Los Del Rio	tropical
2	Thunderstorm	Boris Brejcha	german house, german techno
3	Funkytown	Lipps Inc.	disco, minneapolis sound
4	A Message to You Rudy	The Specials, Rico	punk, ska
5	Lose Control	Missy Elliott, Ciara, Fatman, Scoop	dance pop, hip hop
6	Swagga	Cali Flow Latino	salsa choke
7	Mango Drive	Rhythm & Sound	electro jazz
8	Dont Stop Til You Get Enough	Michael Jackson	pop, r&b
9	Treasure	Bruno Mars	dance pop, pop
10	Tieduprightnow	Parcels	aussietronica, indie soul
11	ZEZE	Kodak Black, Offset, Travis Scott	florida rap, hip hop
12	Make Me Feel	Janelle Monae	afrofuturism, alternative r&b
13	yours truly.	Super Whatevr	alternative emo, anthem emo,
14	Uptown Funk	Mark Ronson, Bruno Mars	dance pop, neo soul
15	You Rock My World	Michael Jackson	pop, r&b
16	People Get Up And Drive Your Funky Soul	James Brown	funk, motown
17	Get Down On It	Kool & The Gang	disco, funk
18	Jump (For My Love)	The Pointer Sisters	dance pop, disco
19	Kill The Lights	Alex Newell, DJ Cassidy, Vinyl on HBO, Nile Rodgers	indie poptimism
20	Always On Time	Ja Rule, Ashanti	dance pop, east coast hip hop
21	Dang!	Mac Miller, Anderson.Paak	hip hop, pittsburgh rap
22	Faded	Cool Company	alternative r&b, indie r&b
23	Lose Yourself to Dance	Daft Punk, Pharrell Williams	electro, filter house
24	Hasta el Amanecer	Nicky Jam	latin, latin hip hop
25	Shape of You	Ed Sheeran	pop, uk pop
26	Freestyler	Bomfunk MC’s	bubblegum dance, eurodance
27	Hot Stuff	Donna Summer	dance pop, disco
28	Magalenha	Sergio Mendes	bossa nova, brazilian jazz
29	24K Magic	Bruno Mars	dance pop, pop
30	Turn Down for What	Dj Snake, Lil Jon	dance pop, edm

List of 30 songs named by 30 participants that they want to move to which is ordered according to the highest Spotify danceability scores from the dance music dataset in descending order. Only the first two genre tags extracted from Spotify are presented in this table.

### Subgroups of dance music

**Fig 2:**
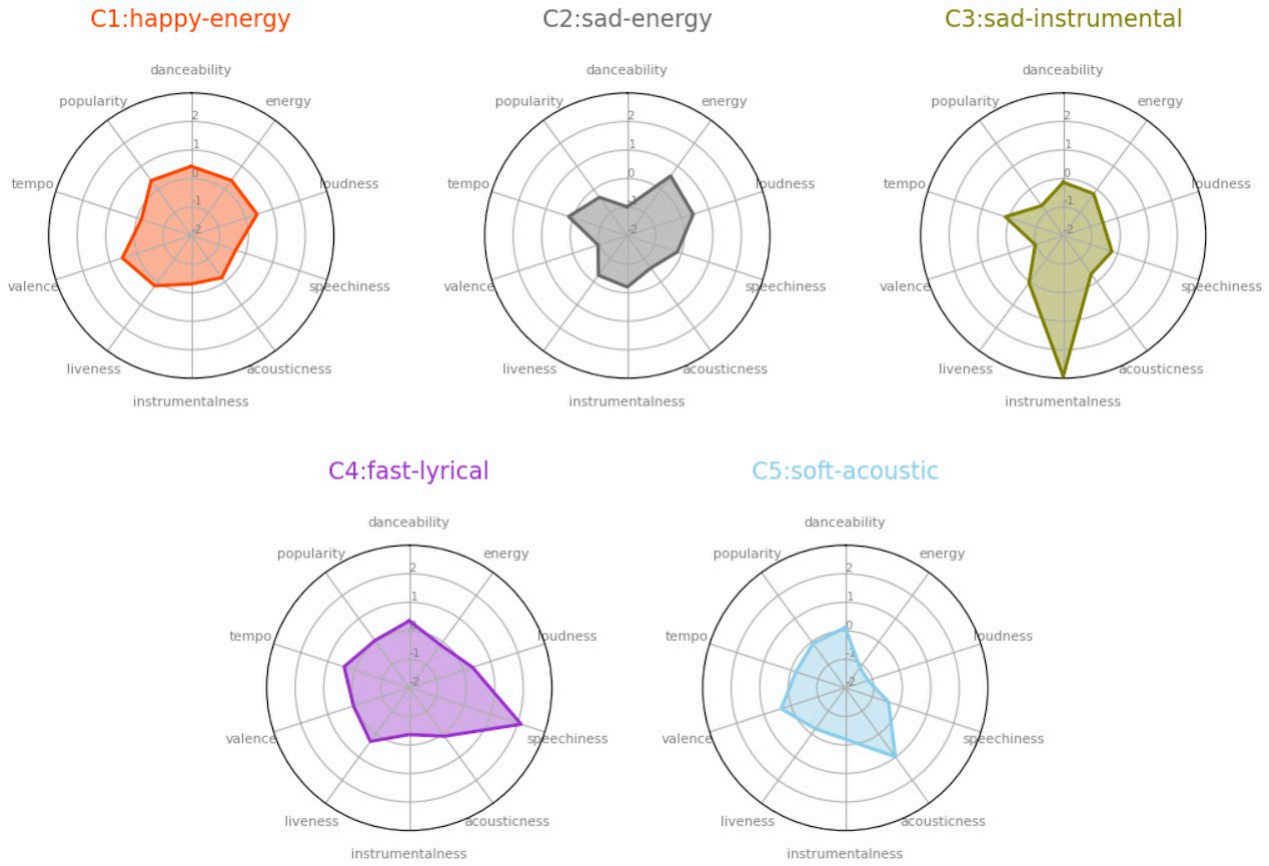
Subgroups of dance music. Five clusters of dance songs (*N* = 278) are illustrated with individual Spotify audio feature variation. Clusters were named as “happy-energy” (*N* = 101), “sad-energy” (*N* = 60), “sad-instrumental” (*N* = 23), “fast lyrical” (*N* = 35) and “soft-acoustic” (*N* = 59) according to their most distinctive audio features. Please note that all of the features are standardised.

To determine whether or not all dance music had similar Spotify audio features, we performed a k-means clustering analysis. The results revealed 5 different subgroups of participants’ dance songs depending on their Spotify audio features. See Fig 2 for a visual representation of this analysis.

*Cluster 1* (C1) consisted of 101 songs characterised by elevated levels of valence, danceability, loudness, and popularity. Songs in this cluster came from a range of genres, in particular pop, rock, dance, hip-hop, rap, soul, funk, and indie (e.g., *Thriller* by Michael Jackson, *Lose Yourself to Dance* by Daft Punk and Pharrell Williams, *Smooth* by Santana and Rob Thomas). *Cluster 2* (C2) consisted of 60 songs characterised by lower levels of danceability, valence, and acousticness, and higher levels of energy and loudness. In this cluster, pop, rock, dance, metal, and house were the most frequent genres (e.g., *Titanium* by Sia and David Guetta, *Under Control* by Calvin Harris, Alesso and Hurts, *Laid to Rest* by Lamb of God, *Sad But True* by Metallica). *Cluster 3* (C3) consisted of 23 songs characterised by higher levels of instrumentalness, and lower levels of valence, popularity, and loudness. House, trance, and pop genres dominated in this cluster (e.g., *Insomnia* by Faithless, *Bravo* by Factor B, *You are My High* by Demon). *Cluster 4* (C4) consisted of 35 songs characterised by higher levels of speechiness and tempo. The most common genres here were pop, rock, and hip-hop (e.g., *I* by Kendrick Lamar, *00*:*00 (Zero O’Clock)* by BTS, *Don’t Stop Me Now* by Queen). *Cluster 5* (C5) consisted of 59 songs with higher levels of acousticness, and lower values of loudness and energy. Pop, jazz, and soul were the most frequent genres in this cluster (e.g., *I Wish I Knew How It Would Feel to Be Free* by Nina Simone, *Sway* by Dean Martin, *Blue Skies* by Doris Day).

As observed, while pop was the reoccurring genre in all of the subgroups of dance music, musical characteristics and genre differed across all 5 clusters. We opted to name the clusters based on their musical characteristics. C1 was named “happy-energy” after its audio features of high valence and energy. C2 and C3 contrasted with C1 in terms of their valence scores. C2 was subsequently named “sad-energy” due to low valence and high energy songs, and C3 was named “sad-instrumental” since it was characterized by high instrumentalness and low valence. C4 contained songs with high speechiness and tempo values, and was thus named “fast-lyrical”. Finally, C5 included songs characterised by high acousticness, and low energy and loudness, and was named “soft-acoustic”.

### Reasons for listening

#### Comparing RL general and dance ratings

**Fig 3:**
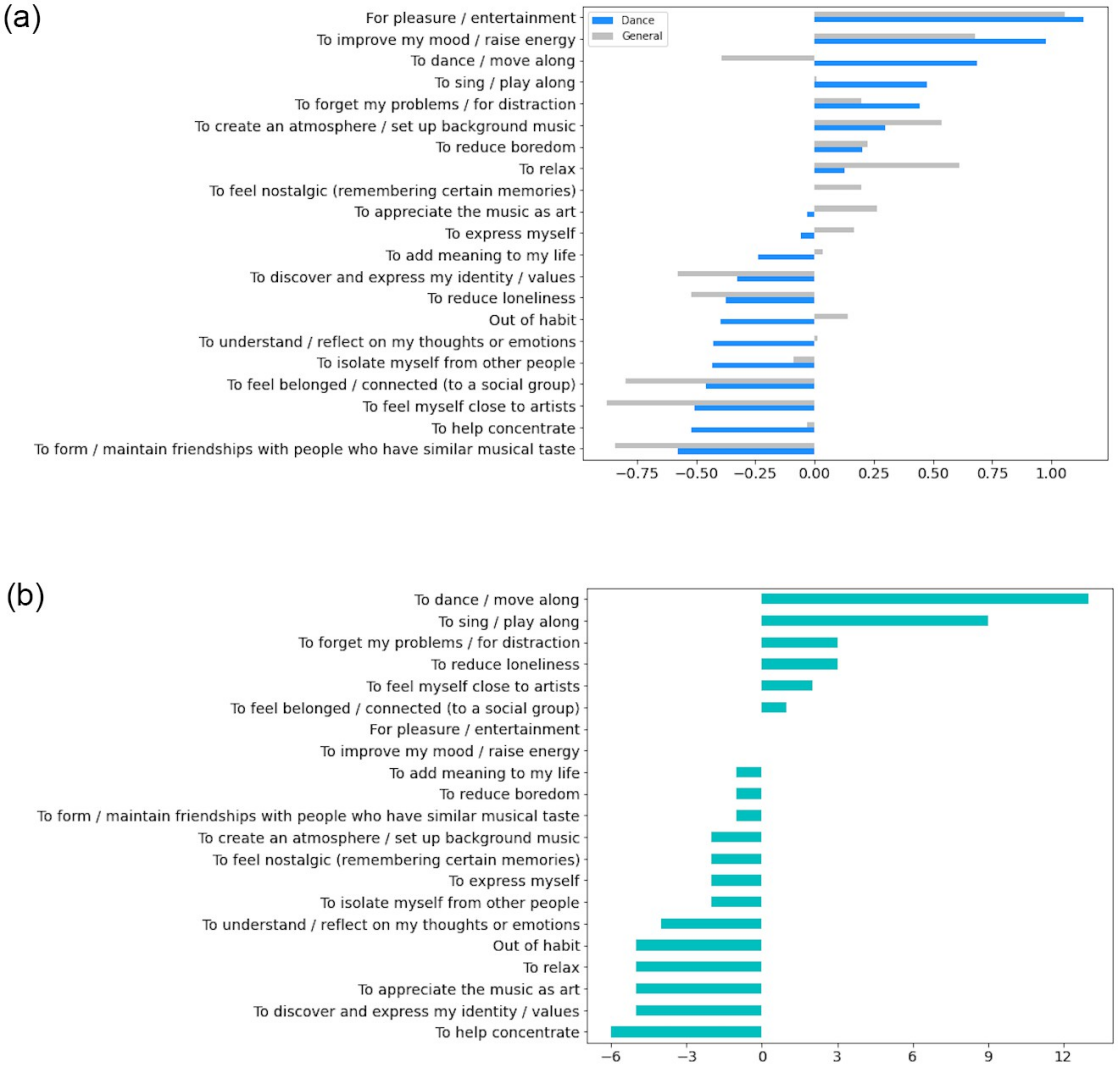
Z-scored distributions of ratings for *RL dance* and *general*. (a) Z-scored frequency distributions of ratings for *RL dance* (blue) and *general* (grey) are plotted. (b) Z-scored rank difference distribution for *RL dance* and *general* ratings. Rank scores of *RL general* items were subtracted from *RL dance* items.

In order to compare *RL general* and *dance*, we z-scored the RL *dance* and *general* ratings and plotted them in the same figure in descending order of RL *dance* ratings (see Fig 3a). It can be seen that, for both dance music and music listening in general, the highest-rated RL were “For pleasure / entertainment” and “To improve my mood / raise energy.” For *RL dance*, the lowest-rated RL were “To form / maintain friendships with people who have similar musical taste” and “To help concentrate”; for *RL general*, the lowest-rated RL was “To feel myself close to artists”. Moreover, whilst the smallest overall difference between *RL dance* and *general* ratings was for “To reduce boredom”, the largest difference was for “To dance / move along”. In other words, people tend to give higher ratings to this item in *RL dance* compared with *RL general*. “To sing / play along” and “To forget my problems” received the next high ratings in *RL dance*. The item “To relax” tended to be rated much higher in *RL* general, indicating greater relevancy for music listening in general compared with a dance related context. Moreover, to illustrate differences between *RL dance* and *general* ratings, the change in rank-ordered RL items from dance to general are plotted in Fig 3b. This figure complements Fig 3a and highlights the following three results. First, it can be seen that the biggest difference in rank order relates to “To dance / move along”, followed by “To sing / play along”. Second, the items “For pleasure / entertainment” and “To improve my mood / raise energy” seem to be equally relevant for both *RL dance* and *general* since there is no change in their rank order for both ratings. Third, the item “To help concentrate” tends to be associated with *RL general* more than *RL dance* since it was ranked 6 places higher in *RL general*.

#### Factor analyses

Next, two separate factor analyses were performed in order to extend the comparison for *RL general* and *dance* ratings. Initially, the results of the two Cattell scree tests [38] revealed the optimum number of factors to be 3 for the *RL general* ratings and 1 for the *RL dance* ratings. Subsequently, the optimum factor numbers were applied in each factor analysis. Table 3 shows the three factor loadings for *RL general*. While the items related to self-awareness and expression such as “To express myself” and “To understand / reflect on my thoughts or emotions” loaded highly on the Factor 1, for the Factor 2 items related to mood and arousal such as “To improve my mood / raise energy” and “To reduce boredom” have high loadings. The Factor 3, on the other hand, is associated with social-related items such as “To form / maintain friendships with people who have similar musical taste” and “To feel belonged / connected (to a social group)”, as well as the items “To sing / play along” and “To dance / move along”. Although one can sing and dance along with the music without anybody being around, such activities are often performed in groups, and the evolution and functions of human dance are frequently discussed as being rather social in nature [42]. Thus, the placement of these items among other social-related items are not surprising. Moreover, the factors explained a total of 47% of the cumulative variance with individual proportions of 18%, 17%, and 13% for the subsequent factors. It’s clear that these are in line with the categorization by Schäfer and colleagues [2]. Thus, we named the emerging factors as *Achievement of self-awareness*, *Regulation of Arousal and Mood*, and *Expression of Social Relatedness*. Since the optimal number factor solution for *RL dance* ratings was 1, the *RL dance* ratings (not factor scores) were used as individual items for the next analysis.

**Table 3:** Factor analysis for the *RL general* ratings.

Reasons for Listening to Music	F1	F2	F3
	Achievement of Self-Awareness	Regulation of Arousal and Mood	Expression of Social Relatedness
To express myself	**.85**	-.13	-.06
To understand / reflect on my thoughts or emotions	**.78**	.10	-.41
To discover and express my identity / values	**.69**	-.22	.25
To add meaning to my life	**.67**	.23	-.06
To appreciate the music as art	**.66**	-.11	.05
Out of habit	**.55**	.14	-.00
To feel nostalgic (remembering certain memories)	**.47**	-.00	.13
To isolate myself from other people	.27	.19	.16
To improve my mood / raise energy	-.07	**.80**	-.05
To create an atmosphere / set up background music	-.13	**.76**	-.00
To reduce boredom	-.11	**.75**	.06
To relax	.21	**.64**	-.05
To forget my problems / for distraction	-.09	**.63**	.19
To help concentrate	.02	**.51**	-.00
For pleasure / entertainment	.30	**.47**	-.08
To reduce loneliness	.25	**.41**	.07
To form / maintain friendships with people who have similar musical taste	-.08	.14	**.84**
To feel belonged / connected (to a social group)	-.13	.12	**.79**
To sing / play along	.18	-.27	**.58**
To dance / move along	-.12	.14	**.57**
To feel myself close to artists	.35	-.06	**.53**

Factor analysis revealed three main functions for the *RL general* ratings. Items with high loadings (> .40) are shown as bold.

### Relating RL to subgroups of dance music

**Fig 4:**
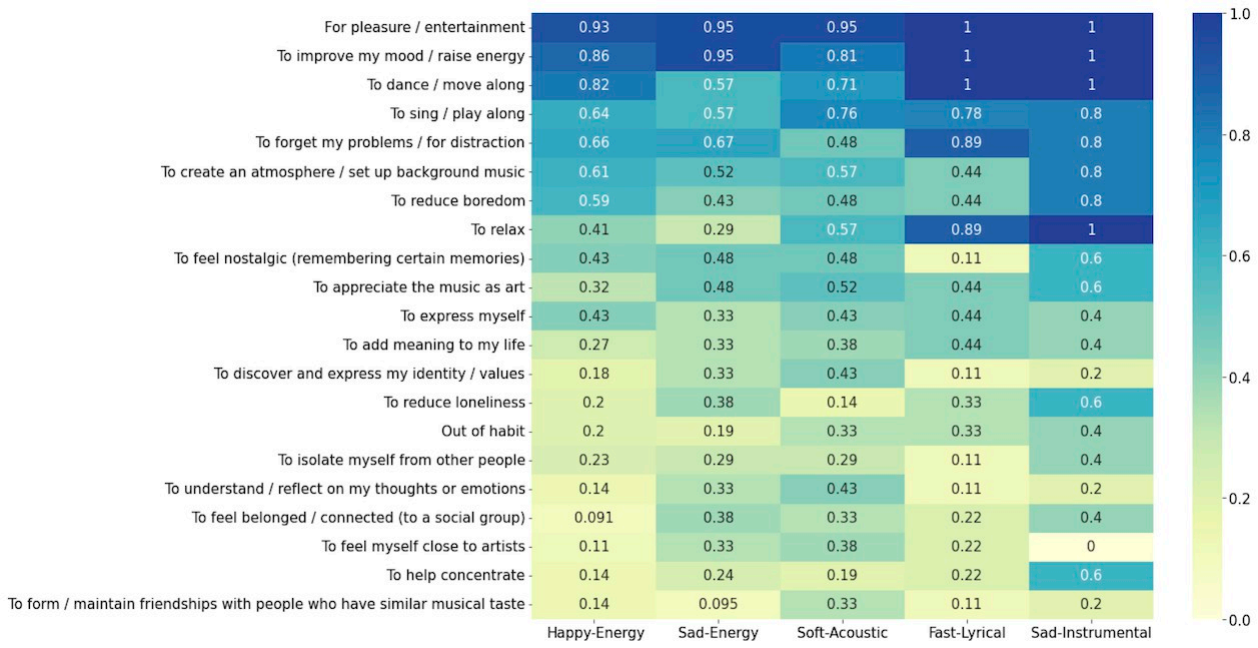
The distribution of participants’ ratings of individual RL items associated with their subgroups of dance music. To relate subgroups of dance music with RL, ratings of the individual *RL dance* items were plotted as percentages for each of the 5 subgroup of dance music (*N* = 100) as a heatmap.

The final research question concerned relationships between musical characteristics of dance music and people’s RL. Fig 4 shows the distribution of participants’ ratings of individual RL items associated with their subgroups of dance music. As predicted, there are variations in the music that people dance to (subgroups of dance music) associated with their particular listening purpose. This suggests that specific subgroups of dance music might be more suitable for specific RL. In the discussion section this figure is interpreted in detail.

## Discussion

We investigated the relationships between Spotify audio features of dance music with associated reasons why people listen to these songs. Specific research questions concerned (1) general audio characteristics of dance music, (2) possible audio feature-based subgroups of dance music, (3) particular RL to dance music and (4) relationships between subgroups of dance music and dance-related RL. In what follows, we discuss our findings.

### General characteristics of dance music

As regards audio characteristics of dance music, the results appear logical: Songs that are typically around 120 bpm, loud, energetic, positively valenced, and score highly on Spotify’s own danceability metric are likely more inviting for listeners to engage with via their body.

While both of the datasets are centred around tempi close to 120 bpm, the baseline dataset’s distribution is rather flat, revealing a wide range of tempi. In contrast, the dance sample has a higher peak around the centre. Interestingly, the shape of the tempo distribution for dance music appears very similar to an earlier study by Moelants [43]. Previous research showed that 120 bpm is not only described as the most efficient locomotion frequency [44] but also mentioned as key to human motion [45]. One other study on music preferences (based on tempo) reported that while engaged in physical exercise, people tend to pick higher tempo ranges compared with slower (95–100 bpm) [46]. In a similar vein, most of the dance songs were reported to particularly be around 120 bpm [47] which is in alignment with the typical speed of human motion, 2 Hz per second [48]. These findings would explain why tempo distribution of dance music (shown in Fig 1i) centred more strictly around 120 bpm with lower variation compared with the baseline dataset. Thus, considering various ways and contexts that music is listened to in general, a higher variation in tempo is expected for music in general.

### Subgroups of dance music

The clustering analysis identified five distinct audio feature-based subgroups of dance music. Songs from these subgroups might be preferred depending on several variables, including as contextual and psychological. Specific examples follow. The “happy-energy” subgroup might be in general associated with popular or mainstream music played, for instance, on TV, radio, and in shopping centres, i.e., music familiar to many people. In contrast with this first subgroup, especially along the valence dimension, the second, “sad-energy” subgroup includes the rock and metal songs. Consisting of sixty tracks, this subgroup was the second largest in our dance music dataset. At first glance, the association of metal and rock music with dance might appear surprising. The demographics and musical preferences of our participants, however, may help explain this relationship. This is because the majority of our data was provided by Finnish participants, and Finland has one of the highest prevalence of metal bands in the world [49]. Thus, this finding might be reflecting musical preferences of many individuals in our sample. With only twenty-three house and trance like songs, the third “sad-instrumental” subgroup includes the least number of songs. Since these tracks have low popularity values too, sad-instrumental dance songs might be preferred less frequently than the other types of dance music. The last two subgroups, “fast-lyrical” and “soft-acoustic”, also contrast in terms of their Spotify audio features. An example song from the fast-lyrical subgroup is *Don’t Stop Me Now* by the Queen. Listeners might prefer such songs firstly to get energised or match their energy level with the song. The high levels of speechiness or lyrical content of the piece might also allow listeners to express themselves and their emotions. These functions might further engage listeners on a bodily level. Soft-acoustic songs, on the other hand, might serve a slightly different function, and afford different types of engagement. Taking Nina Simone’s *I Wish I Knew How It Would Feel to Be* as an example, one might prefer to listen to this song to feel relaxed, sway along with the song slowly, or dance with a partner possibly romantically as well. Nevertheless, the scope of the current study does not allow us to understand in detail how and why these subgroups are preferred in people’s lives. Yet, listening preferences for specific dance music might vary depending on several factors. Because of this, further research considering context-dependent variables and accounting for individual differences is encouraged.

Previous research has also demonstrated that there are different types of dance music according to a musical analysis [43]. In particular, focusing on genre classification of dance music depending on tempo distribution, Moelants [43] reported five distinct subgroups: (1) “Trance” (as an “uplifting” type of dance music with an average tempo of 141), (2) “Afro-American” (concentrated on hip-hop, R&B and soul, peaking around 95 bpm), (3) “Fast” (consists of old-style rock & roll and hardcore techno, peaking around 150 bpm), (4) “House” (described as the origin of all modern electronic dance music, peaking around 125–130 bpm), and (5) “General” (consists of different substyles, with style specific variations). Overall, there seem to be typical genre and audio feature combinations for each subgroup of dance music. Moreover, it can be said that individuals’ musical preferences and familiarity with music are among the determinants of the music that people move to. This would explain the diversity of songs presented in Table 2. Previous research is aligned with this interpretation too, since taste and familiarity have been found to influence people’s experience of groove [25]. Thus, it can be said that not only musical features (such as tempo variation) but also interpersonal differences play a role in music being movement-inducing. Therefore, it can be challenging (if not impossible) to nominate “songs that everybody moves to” or “the grooviest song” of all time.

### Reasons for listening

To compare general and dance related RL, we initially plotted participants’ RL for dance and general ratings (see Fig 3). While the distributions were described in detail earlier, the overall picture demonstrates that people tend to listen to music associated with dance for pleasure and entertainment, to improve their mood and raise energy, to dance / move and sing / play along, as well as to distract themselves and forget their problems. Moreover, in order to deepen the comparison of *RL dance* and *general*, we further conducted two factor analyses. While the three-factor solution for general music listening echoes previous literature [2], reflecting general functions associated with music listening, the emergent one-factor solution for *RL dance* should be approached with caution. This solution might be interpreted either as there being only one main function of listening to music associated with dancing, or it could be due to the way the question was presented as it left no room for other functions to be revealed with the current method. In other words, because of time constraints and methodological choices, we were unable to investigate possible contextual variation of music preferences associated with movements. Thus, participants rated these dance-related listening reasons according to the only song they exemplified that they move to. A more detailed study design could inform about the possible other functions for listening to music associated with dance music. This suggestion is discussed further under limitations and future directions.

### Relating RL to subgroups of dance music

Results of the factor analysis (regarding *RL general*) could be used further while interpreting the relationship between the subgroups of dance music with *RL dance* ratings. Fig 4 shows a heatmap plotted for each *RL dance* item separated for each subgroup of dance music. Notably, the most highly-rated *RL dance* items (including “For pleasure / entertainment”, “To improve my mood / raise energy”, “To forget my problems / for distraction”, “To create an atmosphere / set up background music” and “To reduce boredom”) have high loadings for “regulation of mood and arousal” and “expression of social relatedness” factors of *RL general* ratings (as shown in Table 3). Notably, this might signal that the primary function of music listening associated with dance is to regulate one’s emotions and arousal level as well as to express social relatedness. However, this interpretation requires further exploration as not all items (such as “to feel belonged / connected to a social group) from the expression of social relatedness factor were rated highly, as seen in Fig 4.

More detailed consideration of specific subgroups of dance music with particular *RL dance* items yields at least five points worth noting. First, the items “For pleasure / entertainment” and “To improve my mood / raise energy” were mentioned for almost all dance music tracks, and might thus be interpreted as the common RL associated with dancing to music. These can be compared to items such as “To help concentrate” and “To form / maintain friendships with people who have similar musical taste”, which might be interpreted as being the least common RL when dancing to music. Second, the item “To dance / move along” received lower ratings for the dance music subgroup “sad-energy”. Participants’ lower rating of this item makes sense considering the low danceability values of songs in this subgroup. Additionally, the item “To relax” also received lower ratings for this subgroup. With characteristics of high energy and loudness, it seems logical that songs in the “sad-energy” subgroup are not used for relaxation purposes. Third, compared to other subgroups, the item “To feel nostalgic” received lower ratings in the subgroup “fast-lyrical”, which is characterised by high tempo and speechiness. It might be interpreted that songs in this subgroup are not really used to evoke nostalgia. Fourth, compared with other clusters, the “happy-energy” subgroup has the highest number of songs, many of them in the pop genre, and they are characterised by audio features such as high danceability and valence. Considering that many songs for dancing are energising and mood-improving, a greater number of participants’ dance songs accumulating in this category would be expected. Finally, due to there being only 5 songs in the “sad-instrumental” subgroup, we refrain from drawing meaningful conclusions. Further research is clearly needed.

### Limitations and future directions

As mentioned above, the main limitation of the research presented in this paper relates to the study design and data collection method. Since data were collected as part of an extensive survey, details about particular situations of the listener and listening experience were not obtained. Future experimental designs which consider current mood, energy level, personal habits, goals, specific situations (such as listening alone or in a group setting, in a club or house party, or while commuting) could shed further light on the occasions when and how often people listen to particular subgroups of music. In particular, we suggest that subsequent work employs an experience sampling method [34, 50]. We believe this would be a reliable and sensitive method for measuring variables that could influence dance-related RL. We might predict that, depending on the variables related to listener, music, and situation, RL associated with dancing would vary. For instance, while listening to a song in a club with your friends could be more related to expression of social connection, listening to the same song the next day while commuting could be associated with nostalgia and self-awareness. Thus, these functions might appear not fixed, but context-dependent.

A second limitation relates to sample size. While previous research on different types of sleep music [30] collected data solely from Spotify, which thus allowed investigation of a substantial sample, the goal in the current study of investigating particular reasons associated with songs that people move to required a questionnaire method which limited the data for dance music to the number of respondents. Especially when *RL dance* ratings were divided according to the subgroups of dance music, the size of some of the subgroups did not allow for further interpretation. Hence, we hope that future research will pay attention to connecting different RL for different dance music types with a bigger sample. Similarly, targeting a larger sample of individuals would enrich our understanding of culture-specific preferences for dance music (as exemplified earlier by Finns’ preferences for rock and metal music) and thus could present a more elaborate representation of music that people use for dancing.

In summary, the research presented in this paper described audio features and reasons for listening associated with songs that people move to. We reported that loud, energetic songs with positive valence, high danceability and a tempo close to 120 bpm primarily work to regulate mood and arousal through bodily movement. At the same time, some variation in Spotify audio features and associated RL was apparent. In addition to more research connecting audio features of dance music with RL, other noteworthy issues concern the extra- and intra-musical prerequisites of certain songs that induce movements, as well as how music-induced movements vary depending on these extra- and intra-musical variations. In other words, what specific features are necessary to make music danceable and how do people’s psycho-physical experiences differ depending on those variables? The relationship between music and movement, particularly the idea of “different types of groove experiences” [17] thus remains open for further discoveries.

## Supporting information

S1 Text Description of Spotify audio features. (DOCX)
