# Supplementary material for: Music we move to: Spotify audio features and reasons for listening
Source: PLoS One. 2022 Sep 29;17(9):e0275228. doi: 10.1371/journal.pone.0275228 (PMC9522267; doi:10.1371/journal.pone.0275228)
Supplement: S1 Text — (DOCX) [file pone.0275228.s001.docx]

**Description of Spotify Audio Features**

Description of Spotify audio features are presented below, which are taken from Web API Reference | Spotify for Developers [1].

- **Acousticness:** A confidence measure from 0.0 to 1.0 of whether the track is acoustic. 1.0 represents high confidence the track is acoustic.
- **Danceability:** Danceability describes how suitable a track is for dancing based on a combination of musical elements including tempo, rhythm stability, beat strength, and overall regularity. A value of 0.0 is least danceable and 1.0 is most danceable.
- **Energy:** Energy is a measure from 0.0 to 1.0 and represents a perceptual measure of intensity and activity. Typically, energetic tracks feel fast, loud, and noisy. For example, death metal has high energy, while a Bach prelude scores low on the scale. Perceptual features contributing to this attribute include dynamic range, perceived loudness, timbre, onset rate, and general entropy.
- **Instrumentalness:** Predicts whether a track contains no vocals. "Ooh" and "aah" sounds are treated as instrumental in this context. Rap or spoken word tracks are clearly "vocal". The closer the instrumentalness value is to 1.0, the greater likelihood the track contains no vocal content. Values above 0.5 are intended to represent instrumental tracks, but confidence is higher as the value approaches 1.0.
- **Liveness:** Detects the presence of an audience in the recording. Higher liveness values represent an increased probability that the track was performed live. A value above 0.8 provides strong likelihood that the track is live.
- **Loudness:** The overall loudness of a track in decibels (dB). Loudness values are averaged across the entire track and are useful for comparing relative loudness of tracks. Loudness is the quality of a sound that is the primary psychological correlate of physical strength (amplitude). Values typically range between -60 and 0 db.
- **Speechiness:** Speechiness detects the presence of spoken words in a track. The more exclusively speech-like the recording (e.g. talk show, audio book, poetry), the closer to 1.0 the attribute value. Values above 0.66 describe tracks that are probably made entirely of spoken words. Values between 0.33 and 0.66 describe tracks that may contain both music and speech, either in sections or layered, including such cases as rap music. Values below 0.33 most likely represent music and other non-speech-like tracks.
- **Tempo:** The overall estimated tempo of a track in beats per minute (BPM). In musical terminology, tempo is the speed or pace of a given piece and derives directly from the average beat duration.
- **Valence:** A measure from 0.0 to 1.0 describing the musical positiveness conveyed by a track. Tracks with high valence sound more positive (e.g. happy, cheerful, euphoric), while tracks with low valence sound more negative (e.g. sad, depressed, angry).

**References**

1. Web API reference: Spotify for developers [Internet]. Home. [cited 2022Sep18]. Available from: https://developer.spotify.com/documentation/web-api/reference/#/operations/get-several-audio-features
